# The Book World of Medicine and Science

**Published:** 1902-10-11

**Authors:** 


### The Book World of Medicine and Science.

The Essentials of Histology, Descriptive and Practical, for the Use of Students. By E. A. Schafer LL.D., F.R.S. (Sixth edition. 1902. Price 9s. net)

This book serves two purposes; on the one hand it is a guide to the microscopical examination of the tissues of the body, and on the other it is descriptive of these tissues, and thus also serves as a text-book of histology. There are, of course, many points in the more abstruse aspects of the science which are not entered into, but the book before us certainly contains all that can possibly be required by the medical student in relation to the subject. After an introductory chapter on the animal cell, which is the basis of all histology, the rest of the book is divided into lessons, each of which is meant to cover approximately the work which may be gone through in from one to three hours, according to the relative extent to which the preparations are made beforehand by the teacher or during the lesson by the students. Beginning with the microscope and its use, we proceed to that most accessible cell the blood corpuscle, and to the other visible constituents of the blood, in man and in the amphibia, their amœboid movements being afterwards studied. Then we come to epithelium, to connective tissue, bone, muscle, nerve, and all the various structures met with in the animal frame, till finally we reach an appendix devoted to formulæ, methods and apparatus. In this, the sixth edition, a good deal of new matter has been added. This is especially noticeable in the part devoted to the central nervous system, but all through one finds evidences of the care which has been taken to render the book a reliable exposition of the present state of the science of histology, and to make the facts of this science intelligible to the student. At every stage the matters described are beautifully illustrated, and these illustrations, which are all intercalated in their proper places in the text, are most admirably printed. Let us not omit also a word of praise for the binding which allows the book to open well and lie flat upon the table, an immense advantage in a volume which is intended to be one's companion while actually working with the microscope.

A Practical Treatise on Small-pox. Illustrated by coloured photographs from life. By George Henry Fox, M.D., with the collaboration of S. D. Hubbard, M.D., S. Pollitzer, M.D., and J. H. Huddleston, M.D. (Philadelphia and London: J. B. Lippincott Co. 1902. Price, complete in two parts, 12s. net.)

These plates illustrating the various phases and varieties of small-pox are issued in form and size similar to those forming the "Photographic Atlas of Diseases of the Skin,' recently published by the same author, which we have already favourably noticed. The letter-press contains a very carefully written account of the disease, special attention being given to those questions of diagnosis which are often so puzzling, and for the elucidation of which the plates are more particularly intended. We can well believe that, as the author says, many of these photographs have been obtained under great difficulty. Some of them are admirable, and serve well to illustrate the very varied appearances presented by the disease. Others are less successful. There is always a difficulty in the employment of photography for depicting skin diseases, where so much depends upon mere variety of tint especially seeing that the play of colour in these diseases lies so largely among the reds and yellows. The result has been that in some of the examples now before us the red areola around the vesicles appears to have become rather exaggerated in depth of tone, and as the artist who has done the colouring seems to have followed suit, the skin has in some of the pictures been made to appear more angry and inflamed than is usually the case at the stages of the disease which they illustrate. This is the only criticism we have to make, and this we are afraid is inherent in photographic processes. Otherwise this work provides a most excellent series of pictures which will, we think, be found of great service by that large number of medical men who, in consequence of the long periods which so often intervene between epidemics of small-pox, can have had very slight opportunities for making themselves acquainted with the varied phases of the disease.

Digest of Public Health Cases. By J. E. R. Stephens, of the Middle Temple, Barrister-at-law. (London: The Sanitary Publishing Company. 1902. Price 21s. net.)

This is a most useful book. To anyone even distantly concerned in public health matters it is full of interesting information, while to those who are officially engaged in the administration of the sanitary law it is sure to become absolutely necessary as a book of reference. An Act of Parliament is one thing, the interpretation thereof is quite another. Statutes appear plain enough; it is when one reads cases that the surprises come in, and so largely does actual sanitary administration depend upon case-law that some such digest as this is quite a necessity to sanitary officials. It is very well done, the cases are stated briefly but sufficiently, and their points are not lost sight of. A careful perusal of a selection from its pages strongly impresses one with the feeling that much trouble has been taken in its compilation and that it is authoritative and trustworthy.

